# DA5-CH and Semaglutide Protect against Neurodegeneration and Reduce *α*-Synuclein Levels in the 6-OHDA Parkinson's Disease Rat Model

**DOI:** 10.1155/2022/1428817

**Published:** 2022-11-14

**Authors:** Lingyu Zhang, Chun Li, Zijuan Zhang, Zhenqiang Zhang, Qian-Qian Jin, Lin Li, Christian Hölscher

**Affiliations:** ^1^Key Laboratory of Cellular Physiology, Shanxi Medical University, Taiyuan, Shanxi, China; ^2^Department of Forensic Pathology, Shanxi Medical University, Taiyuan, Shanxi 030001, China; ^3^Academy of Chinese Medical Sciences, Henan University of Chinese Medicine, Zhengzhou 450046, Henan Province, China; ^4^Second Hospital Neurology Department, Shanxi Medical University, Taiyuan, Shanxi, China

## Abstract

Insulin desensitization has been observed in the brains of patients with Parkinson's disease (PD), which is a progressive neurodegenerative disorder for which there is no cure. Semaglutide is a novel long-actingglucagon-likepeptide-1 (GLP-1) receptor agonist that is on the market as a treatment for type 2 diabetes. It is in a phase II clinical trial in patients with PD. Two previous phase II trials in PD patients showed good effects with the older GLP-1 receptor agonists, exendin-4 and liraglutide. We have developed a dual GLP-1/GIP receptor agonist (DA5-CH) that can cross the blood-brain barrier (BBB) at a higher rate than semaglutide. We tested semaglutide and DA5-CH in the 6-OHDA-lesion rat model of PD. Treatment was semaglutide or DA5-CH (25 nmol/kg, i.p.) daily for 30 days postlesion. Both drugs reduced the apomorphine-induced rotational behavior and alleviated dopamine depletion and the inflammation response in the lesioned striatum as shown in reduced IL-1*β* and TNF-*α* levels, with DA5-CH being more effective. In addition, both drugs protected dopaminergic neurons and increased TH expression in the substantia nigra. Furthermore, the level of monomer and aggregated *α*-synuclein was reduced by the drugs, and insulin resistance as shown in reduced pIRS-1^ser312^ phosphorylation was also attenuated after drug treatment, with DA5-CH being more effective. Therefore, while semaglutide showed good effects in this PD model, DA5-CH was superior and may be a better therapeutic drug for neurodegenerative disorders such as PD than GLP-1 receptor agonists that do not easily cross the BBB.

## 1. Introduction

Parkinson's disease (PD) is a chronic neurodegenerative disorder diagnosed clinically based on the presence of typical motor symptoms and mainly characterized by progressive dopaminergic neuronal loss in the substantia nigra (SN), which leads to striatal dopamine deficiency [1, 2]. The oligomeric and fibrillar forms of *α*-synuclein can affect mitochondria function [3, 4], induce oxidative stress [5], impair the autophagy-lysosome pathway [6], and induce the death of nigrostriatal neurons. In addition, PD has been associated with upregulated neuroinflammation with consequent neuronal loss in distinct brain areas in specific brain areas of the substantia nigra (SN) and striatum [7]. Postmortem studies have further shown evidence of the overproduction of proinflammatory cytokines in the CSF and SN of patients with PD [8, 9]. Although the pathogenic mechanism of PD is not certain, a growing body of epidemiological and clinical data suggests age-related insulin resistance is a risk factor for PD [10–18]. Studies indicate that *α*-synuclein can activate microglia, leading to the release of proinflammatory cytokines [19], which could inhibit downstream insulin signaling and lead to the development of neuronal insulin resistance [20–22]. As insulin acts as a key growth factor in the brain, insulin desensitization can lead to reduced energy utilization, gene expression, cell repair, and autophagy [10, 12, 23–25].

Glucagon-likepeptide-1 (GLP-1) is an incretin hormone that can enhance insulin signaling. Analogues of GLP-1 have been developed as a treatment for type 2 diabetes (T2DM) [26, 27] and to improve insulin signaling in T2DM [28–31]. Importantly, studies demonstrated that GLP-1 receptor agonists have good neuroprotective effects in animal models of PD [32–36]. The first GLP-1 mimetic exendin-4 (Exenatide, Byetta, and Bydureon), brought to the market to treat T2DM, showed a therapeutic effect in preclinical tests of PD [33, 37, 38]. A pilot clinical trial in PD patients (NCT01174810) had shown good effects [39, 40], and a follow-up phase II placebo-controlled clinical trial confirmed the protective effects in PD patients [41, 42]. Another phase II clinical trial showed that the GLP-1 analogue liraglutide (Victoza), which is on the market as a treatment for T2DM, can improve PD pathology (NCT02953665) [42, 43]. That phase II trial testing liraglutide showed good protective effects, improving everyday motor activities, cognition, memory, and quality of life scores [43]. Semaglutide (Ozempic, Rybelsus) is a modification of liraglutide with a much-enhanced survival time in the blood [44] and is being marketed as a once-weekly drug to treat T2DM [29, 45]. It is currently in a clinical phase II trial in PD patients [25]. In our previous study, we demonstrated that semaglutide showed promising protective effects in the MPTP mouse model [46, 47]. The studies show that semaglutide improved behavioral deficits, reversed the decrease of tyrosine hydroxylase (TH) expression, reduced *α*-synuclein levels, and alleviated the chronic inflammation response in the substantia nigra and striatum. However, this drug does not readily enter the brain [48, 49], as it has been designed to remain in the blood stream for a week [30]. A glucose-dependent insulinotropic polypeptide (GIP) is the “sister” incretin hormone of GLP-1 and has neuroprotective effects on its own [50]. We developed a novel dual GLP-1/GIP receptor agonist (DA5-CH) that can cross the BBB readily [51, 52] and shows good neuroprotective effects in the MPTP mouse model [53, 54] and the 6-OHDA rat model [51]. The MPTP mouse model has its limitations and does not display loss of dopaminergic neurons. The present study was designed to examine the neuroprotective effects of semaglutide in direct comparison to DA5-CH using the 6-OHDA-lesion PD rat model, which shows loss of neurons in the SN [55].

## 2. Material and Methods

### 2.1. Reagents and Drugs

Reagents used were 6-hydroxydopamine hydrobromide (6-OHDA, Sigma, USA), pargyline hydrochloride (MedChemExpress, USA), desipramine hydrochloride (MedChemExpress, USA), apomorphine (Absin Bioscience Inc, China), paraformaldehyde (PFA, Boster Biotechnology, China), RIPA lysis buffer (Beyotime Institute of Biotechnology, China), BCA Protein Assay Kit (Boster Biotechnology, China), ECL-enhanced chemoluminescence (Boster Biotechnology, China), Dopamine ELISA kit (Cloud-clone crop, China), TNF-*α* ELISA kit (Cloud-clone crop, China).

Semaglutide had been purchased from Bachem (Switzerland), and DA5-CH was synthetized by China peptides. The purity of each peptide was analyzed by reversed-phase HPLC and characterized using matrix-assisted laser desorption/ionization time of flight (MALDI-TOF) mass spectrometry, with a purity >97%. Peptides were reconstituted in ultrapure water (Milli-Q) to a concentration of 1 mg/ml, and aliquots were prepared and stored at −20°C.

### 2.2. Animals, Stereotactic Surgery, and Drug Administration

All animal experiments were carried out according to the National Institutes of Health Guide for the Care and Use of Laboratory Animals. All experiments were approved by the Shanxi Medical University ethics board. Forty adult male Sprague-Dawley (SD) rats (obtained from the Shanxi Medical University Experimental Animal Center) that were three months old and weighted220–250 g each were used in the present study. All animals had ad libitum access to water and a standard rat diet. They were grouped and housed in the animal room under a 12 h/12 h light-dark cycle, controlled temperature of 23°C ± 2°C and 55% ± 10% humidity.

Thirty minutes prior to stereotactic surgery, rats received pargyline (5 mg/kg; monoamine oxidase inhibitor) and desipramine (10 mg/kg; noradrenaline uptake inhibitor) to protect noradrenergic terminals against 6-OHDA-induced damage and to increase the selectivity of 6-OHDA for dopaminergic neurons. After administration of general anesthesia (Ketamine 100 mg/kg body weight and Xylazine 10 mg/kg body weight) by intraperitoneal injections, rats were fixed in a standard stereotaxic frame (RWD Life Science in Shenzhen, China) and received a single injection of 6-OHDA (2 mg/ml dissolved in physiological saline containing 0.05% ascorbic acid) into the right medial forebrain bundle (MFB) (*A* = −2.2 mm anterior to bregma, *L* = −1.5 mm lateral to bregma, *V* = –8.0 mm ventral to dura) over the course of two minutes. The syringe was left in place for 10 min for diffusion before being slowly retracted back.

**Figure 1:**
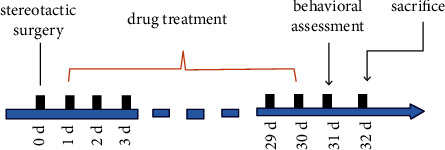
Schematic diagram of the study. The timeline and sequence of treatments conducted are shown.

The rats were randomly divided into 4 groups: a sham + saline group; a 6-OHDA + saline group; a 6-OHDA + semaglutide group; and a 6-OHDA + DA5-CH group, with 10 rats in each group. For the 6-OHDA + drug group, rats were injected intraperitoneal with 25 nmol/kg b.w. drug once every two days for 31 days (see Figure 1). The sham group was given an equal volume of saline.

### 2.3. Apomorphine Rotation Test

To evaluate the functional effects of the lesions, apomorphine-induced rotational behavior was analyzed. The test was conducted thirty days after the surgery, when the rats had achieved complete postsurgery recovery. Rats were given a subcutaneous injection of apomorphine (0.05 mg/kg), placed individually in a square open field (diameter: 75 cm), and tracked using a computer tracking system (EthoVision XT software, Noldus Information Technology, Netherlands) for 30 min. Completed contralateral rotations were recorded by two examiners that were blinded to animal status. Prior to the behavioral test, animals were habituated to the test room overnight.

### 2.4. Estimation of Striatal Dopamine's Level and Inflammatory Mediators Biomarker

Striatal DA was estimated using the Rat Dopamine ELISA kit (USCN Life Science Inc., Wuhan, China). Similarly, TNF-*α* was assayed using a commercial ELISA assay kit (USCN Life Science Inc., Wuhan, China). A rat IL-1*β*solid-phase Sandwich ELISA (Nawah, Egypt) was used for the analysis of IL-1*β*. All procedures were carried out according to the manufacturers' instructions. In brief, samples (diluted 1 : 2) were added in triplicate to the microtiter plate wells coated with the antibody specific to DA or TNF-*α*, and the plates were incubated for 2 h at 37°C. After removing the unbound substances from each well, a biotin-conjugated antibody was added to the wells, and the plates were incubated for 1 h at 37°C. After washing, wells were incubated with an avidin-conjugated HRP for 30 min at 37°C. Finally, a stop solution was added to stop the reaction, and the color changes were quantified at 450 nm using the BioTek ELisa Reader ELx808. The results were expressed as pg/mg protein.

### 2.5. Western Blotting

Brain tissues were homogenized and lysed in an ice-cold RIPA lysis buffer (Beyotime, China) containing phenyl-methylsulfonyl fluoride (PMSF). The lysates were centrifuged at 12,000 rpm for 20 min at 4°C, and the protein concentration of the total lysates was determined using the BCA Protein Assay Kit (Boster, China). Equivalent amounts of protein were electrophoresed on 12% SDS-PAGE gels and transferred to polyethylene difluoride (PVDF) membranes, which were incubated with primary antibodies in a blocking solution (5% BSA) overnight at 4°C. Nonspecific binding was blocked by incubating the membranes with 5% bovine serum albumin for 1 hour. Proteins were visualized using ECL-plus kits (Boster, China). The blots were made visible using a chemiluminescent imaging system (Sage creation, China), and a densitometric analysis was also run in order to quantify each band.

The following primary antibodies were used: anti-Tyrosine Hydroxylase antibody (1 : 500; Abcam, UK), anti-*α*-Synuclein antibody (1 : 500; Cell Signaling Technology, USA), anti-IRS-1 antibody (1 : 500, Bioworld, USA), and anti-IRS-1(phospho-S312) antibody (1 : 500, Bioworld, USA). Anti-GAPDH (1 : 2000; Bioworld, USA) or rabbit anti-*β*-actin (1 : 2000; Bioworld, USA) served as an internal loading control. The membranes were incubated with secondary antibodies (1 : 5000; Boster, Wuhan, China) subsequently at room temperature for 1 h and were then imaged with the ECL-enhanced chemiluminescence (Beyotime, Shanghai, China). Western blot images were captured with a chemiluminescent imaging system (Sagecreation, Beijing, China), and band intensities were quantified by optical densitometry using Image-Pro Plus 6.0 (Media Cybernetics, USA).

### 2.6. Perfusion and Immunofluorescence

Thirty-two days postsurgery, rats were sacrificed and transcardially perfused with a 4% paraformaldehyde solution (PFA). After overnight postfixation in the same fixative, the brains were transferred to 30% sucrose in PBS for cryoprotection. Then, the brains were snap-frozen and cut at a 40 *μ*m section thickness on a Leica freezing microtome. The substantia nigra pars compacta (SNpc) sections and the striatum sections were cut according to the rat brain Atlas by George Paxinos and Charles Watson (sixth edition). Sections were collected and stored at 4°C.

For immunohistochemistry, sections were moved to 5% H_2_O_2_ in PBS for 15 min to quench endogenous peroxidase activity. After washing, sections were sequentially treated with 0.25% Triton-X 100 in PBS, followed by blocking with 5% goat serum for 1 h and 30 min. They were then immunostained with specific antibodies overnight at 4°C, the primary antibodies being as follows: rabbit anti-TH (Abcam, 1 : 200). Sections were then incubated with fluorescent-labeling goat antirabbit secondary antibody (Boster, 1 : 200) for 2 hours in the dark at room temperature after washing in PBS twice. Finally, sections were viewed by fluorescence microscopy (Olympus BX51, Japan).

### 2.7. Statistical Analysis

Statistical analysis was performed using GraphPad Prism 9.0 software, and data were expressed as the mean ± standard error (SEM). Differences among all groups were determined using a one-way analysis of variance (ANOVA) followed by a Tukey multiple comparison test. *p* < 0.05 was considered statistically significant.

## 3. Results

### 3.1. Both Drugs Normalize the 6-OHDA and Apomorphine-Induced Rotational Behavior with DA5-CH Being More Effective

**Figure 2:**
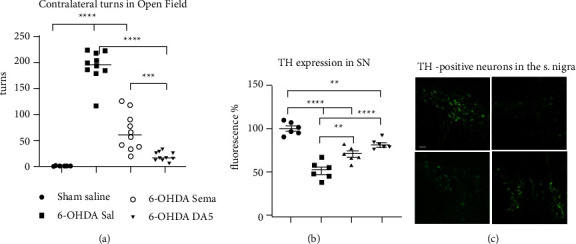
Apomorphine-induced circling and histology of the SN. Animals received apomorphine i.p. (0.05 mg/kg body weight). After 10 min of injection, the rats were placed in an open-field arena, and their activity was recorded for 30 min. (a) Contralateral turns of apomorphine-induced rotation (means and SEM shown, *N* = 10 per group; one-way ANOVA followed by Tukey comparisons test). (b) Quantification of TH labeling in the SNpc. The fluorescence of the sham group was set to 100%. ^*∗∗*^=*p* < 0.005; ^*∗∗∗*^=*p* < 0.001; ^*∗∗∗∗*^=*p* < 0.0001. *N* = 6 per group. (c) Representative fluorescence microscopy images of TH staining in the substantia nigra (scale bar = 50 *μ*m). Top left panel: Sham control; top right panel: 6-OHDA + Sal; lower left panel: 6-OHDA + Sema; lower right panel: 6-OHDA + DA5-CH.

Rotational behavior was tracked to measure the functional recovery of the lesioned brain hemisphere.  Figure 2(a) shows a one-way ANOVA that found a difference across all groups (*F*_3,36_ = 129.2; *p* < 0.0001). Assessment of apomorphine-induced rotation showed that animals in the sham + saline group rats did not exhibit contralateral rotations when challenged with apomorphine (2.3 ± 1.3 net contralateral turns). In posthoc tests, the 6-OHDA + saline group (*p* < 0.0001) and the 6-OHDA + semaglutide (*p* < 0.0001) group showed a clear increase in contralateral rotations after apomorphine injection, when compared with the sham + saline group. When comparing the sham + saline group with the 6-OHDA + DA5-CH group, there was no difference (*p* > 0.05). On the other hand, the 6-OHDA + semaglutide group was significantly different from the 6-OHDA + saline group (*p* < 0.001). The 6-OHDA + DA5-CH group was significantly different from 6-OHDA + saline group (*p* < 0.001) and different from the 6-OHDA + semaglutide group (*p* ≤ 0.0004). The study showed that DA5-CH was more potent than semaglutide in protecting the brain from 6-OHDA toxicity. The sample size was *n* = 10 in each group.

### 3.2. Semaglutide and DA5-CH Protect Dopaminergic Neurons in the Substantia Nigra against 6-OHDA

The analysis of TH levels is usually employed to estimate the reduction of dopaminergic neurotransmitter synthesis, since TH is the enzyme that catalyzes the formation of L-DOPA, which is the rate-limiting step in the synthesis of dopamine. Thus, the number of TH-positive neurons is critical for dopamine synthesis. Figure 2(b) shows a one-way ANOVA found an overall difference between groups (*F*_3,20_ = 36.60, *p* < 0.0001). There was a loss of TH-positive neurons in the SNpc after 6-OHDA + Sal and the 6-OHDA + semaglutide group compared to the sham + saline animals (*p* < 0.0001). Higher TH-positive neurons in the SNc were observed in the DA5-CH treated group compared to the sham + saline animals (*p* < 0.005). There was a difference between the 6-OHDA + saline group and the 6-OHDA + semaglutide group (*p* < 0.005) and the 6-OHDA + DA5-CH group (*p* < 0.0001) (Figure 2(b)). *N* = 6 in each group. Sample histograms are shown in Figure 2(c).

### 3.3. DA5-CH Is More Effective than Semaglutide on Improving the 6-OHDA-Induced Reductions in Striatal Dopamine Content

**Figure 3:**
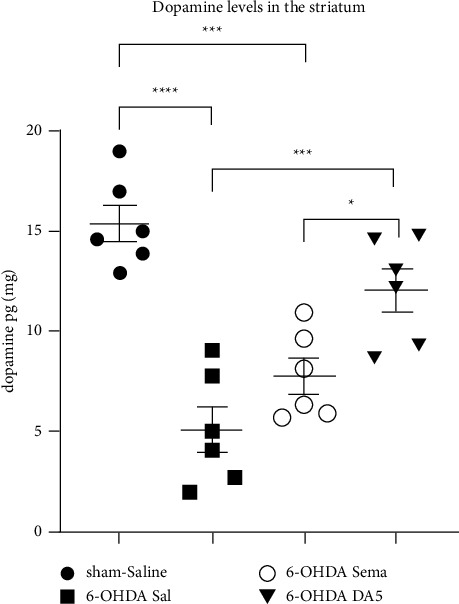
Effects of both drugs on 6-OHDA-induced reduction in striatal dopamine content. The means ± SEM of six rats per group is shown. A one-way ANOVA followed by the Tukey multiple comparisons test;^*∗*^=*p* < 0.05; ^*∗∗∗*^=*p* < 0.005; ^*∗∗∗∗*^=*p* < 0.0001

The recovery of dopamine production in the SN was tested by measuring dopamine levels. A one-way ANOVA found a statistical change overall (*F*_3,20_ = 20.64, *p* < 0.0001). Administration of 6-OHDA caused a prominent decline in striatal dopamine content to the control value (*p* < 0.0001). The 6-OHDA + semaglutide dopamine content was still significantly different from the sham + saline group (*p* ≤ 0.0002) and was not significantly increased compared to the 6-OHDA + saline group (*p* > 0.05). However, the 6-OHDA + DA5-CH dopamine content was not significantly different from the sham + saline group (*p* > 0.05) and was significantly increased compared to the 6-OHDA + saline group (*p* ≤ 0.0002). The 6-OHDA + DA5-CH group dopamine value was higher than the 6-OHDA + semaglutide group value (*p* < 0.05). DA5-CH was more effective in protecting dopamine synthesis than semaglutide (Figure 3). The sample size was *n* = 6 in each group.

### 3.4. DA5-CH Was Superior to Semaglutide in Reducing the 6-OHDA-Induced Increase in Striatal TNF-*α* and IL-1*β* Levels

**Figure 4:**
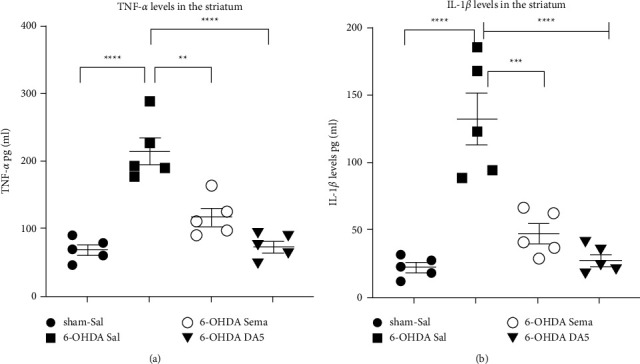
Effects of both drugs on the 6-OHDA-induced increase in striatal TNF-*α* and IL-1*β* content. The means ± SEM of 5 rats per group is shown. A one-way ANOVA followed by the Tukey multiple comparisons test; ^*∗∗*^=*p* < 0.01; ^*∗∗∗*^=*p* < 0.005; ^*∗∗∗∗*^=*p* < 0.0001.

A chronic inflammatory response was induced after a stereotactic injection of 6-OHDA. The effects of drugs on inflammation were tested by measuring proinflammatory cytokines. Figure 4(a) shows a one-way ANOVA found a difference between all groups in TNF-*α* levels (*F*_3,16_ = 26.14; *p* < 0.0001). In posthoc Tukey analyses, as denoted by the elevation of the levels of proinflammatory TNF-*α* in the 6-OHDA + Sal group striatum as compared to the sham + saline group (*p* < 0.0001). Administration of semaglutide reverted such increments of TNF-*α* compared to the sham + saline animals (ns). DA5-CH also reverted the increase compared to the sham + Sal group (ns). However, the 6-OHDA + Sema group showed lower levels compared to the 6-OHDA + Sal (*p* ≤ 0.005), while the 6-OHDA + DA5 group showed even lower levels compared to the 6-OHDA + Sal group (*p* < 0.0001) (Figure 4(a)). The sample number was *n* = 5 in each group.

Figure 4(b) shows that, for the IL-1*β* analysis, a one-way ANOVA found a difference between all groups in IL-1*β* levels (*F*_3,16_ = 22.45; *p* < 0.0001). In posthoc Tukey analyses, the levels of proinflammatory IL-1*β* in the 6-OHDA + Sal group striatum were higher than in the sham + saline group (*p* < 0.0001). Administration of semaglutide reverted such increments of IL-1*β* compared to the sham + saline animals (ns). DA5-CH also reverted the increase compared to the sham + Sal group (ns). However, the 6-OHDA +  Sema groups showed lower levels compared to 6-OHDA + Sal (*p* < 0.0002), while the 6-OHDA + DA5 showed even lower levels compared to the 6-OHDA + Sal group (*p* < 0.0001) (Figure 4(b)). The sample number was *n* = 5 in each group. In conclusion, DA5-CH was more effective in reducing the levels of TNF-*α* and IL-1*β* in the striatum. Administration of semaglutide only partially reverted the increase of TNF-*α* and IL-1*β* observed in the 6-OHDA + Sal group, whereas DA5-CH had a stronger effect.

### 3.5. Both Drugs Protect Dopaminergic Neurons against Enhanced 6-OHDA-Induced*α*-Synuclein Expression with DA5-CH Being Superior

**Figure 5:**
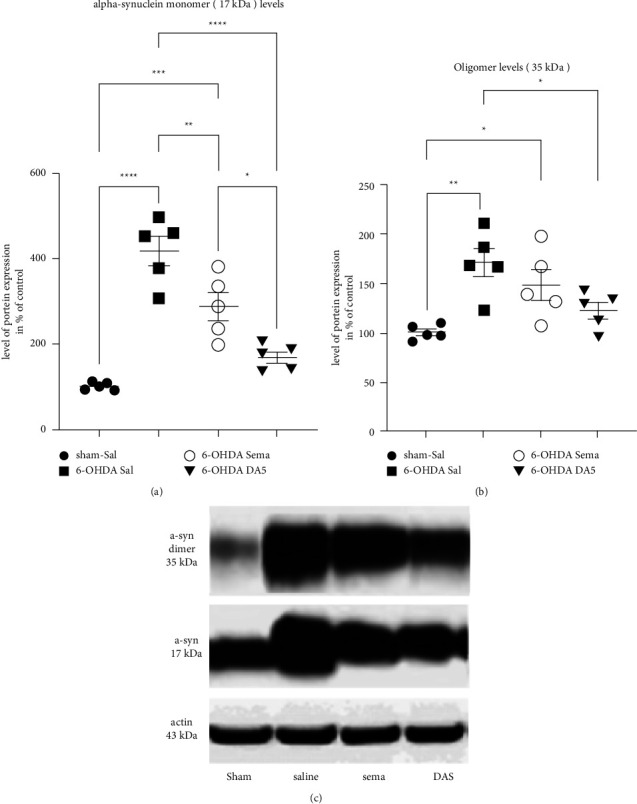
Effects of drugs on 6-OHDA-induced changes in *α*-synuclein expression in the substantia nigra. (a) Analysis of *α*-syn monomers (b) and oligomers expression in SNpc. Each group represents the means ± SEM of 5 rats per group; (one-way ANOVA followed by the Tukey multiple comparisons test*;*^*∗*^=*p* < 0.05*;*^*∗∗*^=*p* < 0.01*;*^*∗∗∗∗*^=*p* < 0.0001*;*^*∗∗∗*^=*p* < 0.001. The legend relates to both (a) and (b) graphs. (c) Sample western blot. Sham-Sal;6-OHDA + Sal;6-OHDA + Sema; 6-OHDA + DA5-CH.

The effect of the drugs on *α*-synuclein expression was measured.  Figure 5(a) shows a one-way ANOVA showed a difference between groups (*F*_3,16_ = 31.74; *p* < 0.0001). *α*-syn protein expression in the SN demonstrated a significant increase in *α*-syn monomer levels compared to the control group (*p* < 0.0001). Compared to the Sham-Sal group, after treatment with semaglutide, *α*-syn monomers levels were still higher in the 6-OHDA + Sema group (*p* < 0.004). However, there was no difference between the Sham-Sal group and the 6-OHDA + DA5 group (ns). There was a difference between the 6-OHDA + Sal and the 6-OHDA + Sema group (*p* < 0.01). The difference between the 6-OHDA + Sal and the 6-OHDA + DA5 groups was higher (*p* < 0.0001). Importantly, there was a significant difference between the 6-OHDA + Sema and the 6-OHDA + DA5 group, demonstrating that DA5-CH was more effective (*p* < 0.05) (Figure 5(a)). The sample number was *n* = 5 in each group.

A one-way ANOVA found a difference between all groups in the *α*-syn oligomers expression levels (*F*_3,16_ = 7; *p* ≤ 0.003). Posthoc tests showed a difference between Sham + Sal and 6-OHDA + Sal (*p* < 0.005) and a difference between Sham + Sal and 6-OHDA + Sema (*p* < 0.05). There was a difference between 6-OHDA + Sal and the 6-OHDA + DA5 (*p* < 0.05) (Figures 5(b) and 5(c)). Sample number was *n* = 5 in each group.

### 3.6. DA5-CH Protects Dopaminergic Neurons against 6-OHDA-Induced Insulin Resistance More than Semaglutide

**Figure 6:**
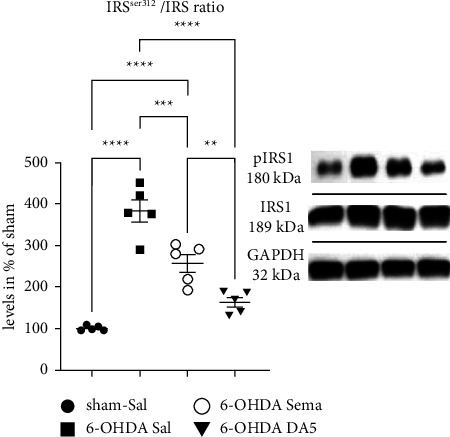
Effects on 6-OHDA-induced insulin resistance in the substantia nigra. Analysis of the IRS/p-IRS-1^ser312^ expression ratio in SNpc. The data represent the means ± SEM of 5 rats per group; one-way ANOVA followed by the Tukey multiple comparisons test; ^*∗∗∗∗*^=*p* < 0.0001; ^*∗∗∗*^=*p* < 0.001; ^*∗*^=*p* < 0.05. Western blot samples are shown. Lane 1: Sham; 2: 6-OHDA; 3: 6-OHDA + Sema; 4: 6-OHDA + DA5.

IRS-1 acts as a critical link in the insulin signaling pathway and can determine the level of insulin sensitivity. In this study, p-IRS-1^ser312^ expression in the SN was measured, and a one-way ANOVA found a difference between groups (*F*_3,16_ = 45.34; *p* < 0.0001). The results demonstrated that compared to the control group, the p-IRS-1^ser312^/IRS-1 ratio increased in rats given 6-OHDA + Sal (*p* < 0.0001). In addition, a reduction in the p-IRS-1^ser312^/IRS-1 ratio in the SNc of PD rats that received DA5-CH was observed, and there was no significant difference compared to Sham-Sal rats (ns). When comparing the 6-OHDA + Sal group to the 6-OHDA + Sema group, there was a clear difference (*p* < 0.001). However, the difference between the 6-OHDA + Sal and the 6-OHDA + DA5 groups was higher (*p* < 0.0001). There was a difference between the 6-OHDA + Sema and the 6-OHDA + DA5 groups (*p* < 0.05) (Figure 6). *N* = 5 in each group.

## 4. Discussion

In several epidemiological studies, diabetes was found to be a risk factor for developing PD [13–16, 18]. One study showed an association between insulin resistance and an increased risk of PD dementia, a more severe PD phenotype [56]. Several other studies found insulin desensitization in the brains of PD animal models regardless of whether they were diabetic or not [57, 58]. Based on this observation, drugs used for the treatment of T2DM targeting insulin resistance have been tested in animal models of PD and in first clinical trials [10, 35]. Importantly, a phase II clinical trial testing the GLP-1 receptor agonist exendin-4 showed protective effects in PD patients. Motor activity was improved compared to the placebo, and the improvement remained visible 3 months after washout. DAT-PET brain imaging demonstrated partial protection of the dopaminergic nigral-striatal projection [41]. A second phase II trial testing the GLP-1 analogue liraglutide showed clear improvements in everyday motor activities such as walking, talking, getting dressed, and getting out of a chair [43]. We have previously shown that GLP-1 receptor agonists can reverse the impairments observed in the MPTP mouse model of PD [12]. Semaglutide is a modified human GLP-1 peptide that is protease-resistant, has two amino acid substitutions compared to human GLP-1 (Aib [8], Arg [34]), and is derivatized at lysine 26. It has an extended spacer for an attached C20 fatty acid for enhanced survival in the blood by binding to albumin [59]. It is on the market as a treatment for diabetes [29, 45]. A phase II clinical trial testing semaglutide in PD patients started in 2019 (NCT03659682), and two phase III clinical trials started in 2021 testing the drug in Alzheimer patients (NCT04777396 and NCT04777409).

Semaglutide and other GLP-1 receptor agonists have been developed to treat diabetes and to remain in the blood stream for extended periods of time. A drawback of this strategy is that these drugs show only limited transfer across the BBB to enter the brain [48, 49, 60]. We and others have shown that in order to treat CNS diseases, these drugs need to be able to enter the brain. There is a direct correlation between entering the brain and protecting the brain from toxic events [49, 53, 61, 62]. We, therefore, developed a novel dual GLP-1/GIP receptor agonist that can cross the BBB at an enhanced rate [12, 54, 62]. This dual agonist, DA5-CH, has shown superior neuroprotective effects in the MPTP mouse model of PD compared to liraglutide [53, 54] and is superior to exendin-4 in the 6-OHDA rat model of PD [51]. Both drugs have shown impressive effects in clinical trials in PD [41–43]. As semaglutide is in clinical trials for AD and PD, we therefore wanted to test this drug side-by-side with DA5-CH.

The 6-OHDA unilateral lesion MBF rat model displays motor deficits consistent with a PD phenotype and furthermore develops a significant loss of TH-positive neurons in the nigrostriatal system to mimic PD [63]. We have previously tested semaglutide in the MPTP mouse model of PD, but this model does not display extensive neuronal loss in the SN [47]. Thus, in order to evaluate the neuroprotective potential of semaglutide, it was necessary to test this drug in the 6-OHDA lesion PD rat model, which displays extensive dopaminergic neuronal loss [51, 63, 64].

Striatal DA depletion is caused by dopaminergic neuronal death and is considered to be a characteristic pathological feature of PD. Rotational behavior has generally been applied to estimate DA depletion following unilateral administration of neurotoxins such as 6-OHDA [64]. In the present study, semaglutide reduced the circling behavior when apomorphine activated dopamine receptors, which showed that the lesioned hemisphere was functional again. In addition, we found semaglutide and DA5-CH rescued neurons in the SNpc from 6-OHDA-induced toxicity and increased the level of striatal DA in PD rats, which demonstrates that both drugs can ameliorate DA depletion from 6-OHDA toxicity. The BBB-penetrating DA5-CH peptide was superior in protecting neurons and improving dopamine levels.

Chronic inflammation in the brain has been shown to be a key feature in PD progression [65, 66]. Initial neuronal damage can activate striatal microglial cells, which release proinflammatory cytokines, leading to a neuroinflammatory process that will end in the neuronal death of vulnerable neuronal populations [67]. Importantly, proinflammatory cytokines drive insulin desensitization and the general reduction of growth factor synthesis and signaling [21, 68–70]. Our data demonstrate that semaglutide and DA5-CH downregulate TNF-*α* and IL-1*β* levels in the striatum of 6-OHDA lesioned rats. This result confirms our previous study findings in the MPTP mouse model of PD, where chronic inflammation in the brain was much reduced by semaglutide [46, 47] and DA5-CH [51, 53, 62]. The reduction of the inflammatory response by GLP-1 receptor agonists is due to the fact that glia cells express the GLP-1 receptor and that GLP-1 acts as an anti-inflammatory cytokine [71, 72]. The reduction of TNF-*α* and IL-1*β* levels in the brain will contribute to the reversal of insulin desensitization that we observed in the 6-OHDA lesioned rats. TNF-*α* can reduce IRS-1 serine phosphorylation and thus inhibits its function [73]. According to our data, the treatment with semaglutide or DA5-CH generally reduced the tyrosine 312 phosphorylation of IRS-1 in 6-OHDA lesion rats. This demonstrates that the drugs could reactivate the insulin signaling pathway, which was blocked by 6-OHDA treatment. Other studies have shown insulin resensitization with GLP-1 receptor agonists [74–77]. The mechanism is most likely driven by the activation of phosphatases that will reactivate IRS-1 and also by the increased gene expression of IRS-1 and insulin receptors [68].

It is postulated that a key feature in the pathology of PD is the aggregation of *α*-synuclein, a component of Lewy bodies in FTD patients [78]. The mechanisms underlying accumulation and aggregation of *α*-synuclein are considered to be overexpression and failure to clear the protein by proteolysis and autophagy [79, 80]. In addition, aberrant forms of *α*-synuclein, including oligomers and fibrils, are seen to interfere with normal cellular processes, promoting further aggregation of protein, leading to the spread of these toxic forms and ultimately leading to neuronal death [81–83]. The cerebrospinal fluid (CSF) of patients with PD contains increased levels of *α*-synuclein oligomers compared to age-matched controls [84]. However, other studies could not detect these *α*-synuclein oligomers in histological sections. They found that while *α*-synuclein oligomers are neurotoxic, Lewy bodies, the fibrillar form, may actually be neuroprotective [85]. The potential mechanisms of *α*-synucleinoligomer-induced neurodegeneration in PD may include the disruption of a variety of cellular processes, such as mitochondrial impairments, endoplasmic reticulum (ER) stress, dysfunction of the autophagy lysosome pathway, and activating microglia to induce the inflammatory response [86]. In our study, a reduction of the *α*-synuclein monomer and oligomer levels was observed after drug treatment. DA5-CH was more effective in lowering the levels than semaglutide. We have previously shown that in the MPTP mouse model, *α*-synuclein expression is very much increased and that semaglutide [47] and DA5-CH [62] both can reduce the levels in the brain. Here, we confirm that result in the 6-OHDA animal model of PD and extend the findings to aggregated forms of *α*-synuclein. In conclusion, these data support the evidence that semaglutide and DA5-CH protect neurons against 6-OHDA induced toxic effects in the rat. The dual agonist DA5-CH was more effective in all parameters measured. Therefore, the administration of this novel dual GLP-1/GIP receptor agonist is a promising candidate for a new treatment of PD.

## Data Availability

The data used to support the findings of this study are included in the manuscript. All authors contributed significantly to the study and writing of the manuscript.
